# Head of the Bed Down: Paradoxical Management for Paradoxical Herniation

**DOI:** 10.5811/cpcem.2019.4.41331

**Published:** 2019-05-29

**Authors:** Patrick D. Bender, Alisha E.C. Brown

**Affiliations:** University of Washington, Harborview Medical Center, Department of Emergency Medicine, Seattle, Washington

## Abstract

Emergency physicians are well versed in cerebral herniation, pathology that typically results from increased intracranial pressure; however, paradoxical herniation is less common and requires opposing treatments. We describe a case of paradoxical herniation following lumbar puncture in a patient with previous hemicraniectomy. The symptomatology was similar to cerebral herniation from intracranial hypertension and included lethargy, bradycardia, headache, and compression of brain structures on non-contrast head computed tomography. However, contrary to treatment modalities for intracranial hypertension, our management strategy aimed to reverse intracerebral hypotension. Treatment for paradoxical herniation involved increasing intracranial pressure using fluid resuscitation and Trendelenburg positioning. In the intensive care unit our patient received an epidural blood patch and hydration with resolution of his symptoms.

## INTRODUCTION

The trajectory of elevated intracranial pressure and impending brain herniation is a disease process in which emergency physicians (EP) are trained and prepared to manage. Usual management strategies aim to reduce intracranial hypertension and include hypertonic osmotic agents, patient positioning with an elevated head of bed, manipulation of cerebral arterial blood flow through hyperventilation and surgical intervention. However, the process of paradoxical herniation is a lesser-known pathology for which the treatment is the contrary to standard therapies for cerebral herniation. Traditional treatments aimed at lowering intracerebral hypertension will worsen paradoxical herniation,^1^ making this a critical condition for EPs to understand. We present a case of paradoxical brain herniation presenting as a post-lumbar puncture (LP) headache in a hemicraniectomy patient. We highlight the underlying pathophysiology, risk factors, presentation, and emergency department (ED) management.

## CASE REPORT

A 19-year-old man presented to the ED with a persistent headache one week after a LP. His past medical history was significant for a stab wound to his left middle cerebral artery one year prior to presentation with surgical management of his injury requiring a left hemicraniectomy. His post-surgical course was complicated by synthetic skull infection and cranioplasty revision, and he was ultimately discharged without a bone flap. One month after discharge, he underwent LP in neurosurgery outpatient clinic for monitoring of previous fungal meningitis and evaluation of flap swelling. In the days following his LP, the patient began to develop a dull, holocephalic headache associated with photophobia, nausea, and vomiting. His headache was worse with sitting and standing. He denied fevers, weakness, or numbness. His caretaker noted he was more lethargic than usual, and the flap overlying his craniectomy site now appeared sunken. There was no new trauma reported. A week into his symptoms, he was referred to the ED for evaluation of post-LP headache.

Vital signs on presentation to the ED were as follows: heart rate of 45 beats per minute, blood pressure of 93/73 millimeters of mercury, respiratory rate of 14 breaths per minute, and a temperature 37 degrees Celsius. He was drowsy but arousable and could answer simple questions. His presenting Glasgow Coma Scale was 14. Head examination revealed a sunken scalp flap overlaying the left anterior temporal craniectomy site without erythema, drainage or fluctuance. There was no evidence of new cranial trauma. His cranial nerve examination revealed no deficits, with symmetric and reactive pupils. He had full and symmetric strength in his upper and lower extremities with no obvious sensory changes. His cardiac exam was notable for bradycardia. His LP site was well appearing without leakage, erythema, bruising, or tenderness. The remainder of his examination was normal. Cerebrospinal fluid studies from earlier in the week were reviewed and showed zero white blood cells and zero red blood cells.

**Image 1:**
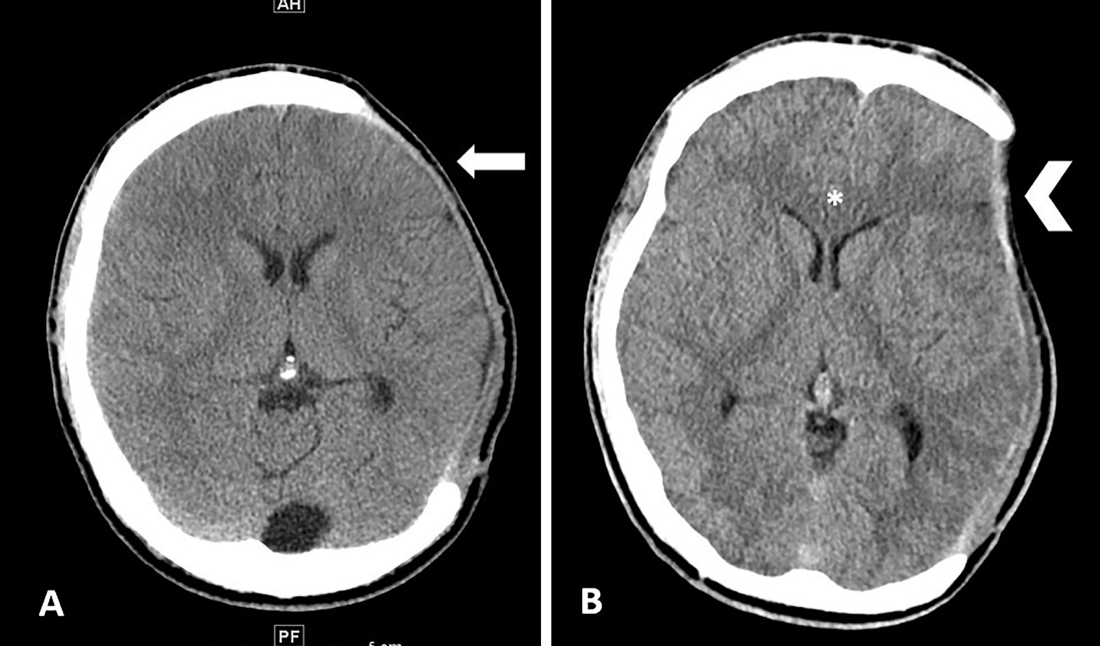
Non-contrast axial head computed tomography (CT) image demonstrating sunken flap and midline shift. Image A is from seven weeks prior to patient’s emergency department (ED) presentation. He is status post left hemicraniectomy. His CT demonstrates a normal-appearing cranioplasty flap (arrow). In comparison, image B is from the day of patient’s ED presentation demonstrating flattening and depression at the area of skull defect (arrowhead) with a corresponding 5 millimeter left-to-right midline shift (star) concerning for paradoxical herniation.

**Image 2:**
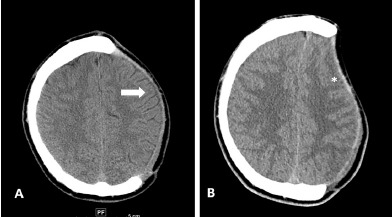
Non-contrast axial head computed tomography (CT) image demonstrating sulcal effacement. Image A is from seven weeks prior to patient’s emergency department (ED) presentation. He is status post left hemicraniectomy. His CT demonstrates a normal-appearing cranioplasty flap (arrow). In comparison, image B is from the day of patient’s ED presentation and demonstrates sulcal effacement (star).

In the ED, the patient underwent a non-contrast head computed tomography (CT), which demonstrated a 5 millimeter (mm) left-to-right midline shift with subfalcine herniation (Image 1), sulcal effacement (Image 2), and soft tissue sinking consistent with paradoxical herniation. We placed the patient’s head of bed down, gave him one liter normal saline, metoclopromide for nausea, and acetaminophen and hydromorphone for pain control. Neurosurgery was emergently consulted and the patient was admitted to the intensive care unit (ICU). He underwent an epidural blood patch with anesthesia the next morning. Repeat non-contrast head CT one day later showed decreased midline shift from 5 mm to 3mm and improved sunken appearance of the left cerebral hemisphere. On hospital day two, his headache had improved and he was discharged in stable condition with plans for outpatient cranioplasty. Follow-up of his cerebrospinal fluid cultures revealed no fungal or bacterial growth.

**CPC-EM Capsule**

What do we already know about this clinical entity?

Paradoxical herniation occurs in the setting of a cranioplasty when atmospheric pressure exceeds intracranial pressure, resulting in compression of critical brain structures.

What makes this presentation of disease reportable?

This is a rare disease entity in cranioplasty patients resulting in cerebral herniation. However, interventions are opposite that of herniation syndromes more commonly encountered in intracranial hypertension.

What is the major learning point?

Forces generating herniation in paradoxical herniation originate from a low pressure system. Management should be aimed at increasing intracranial pressure.

How might this improve emergency medicine practice?

Recognition of important physical exam findings such as a sunken flap may provide early clues to this diagnosis, facilitating timely treatment and appropriate disposition.

## DISCUSSION

Decompressive craniectomy is a neurosurgical procedure in which part of the skull is removed in an effort to prevent brain herniation in the setting of severely elevated intracranial pressure. It is typically performed in patients with life-threatening traumatic brain injury,^2^,^3^ or extensive stroke.^4^ Once the primary neurologic insult has improved or resolved, most patients will undergo a cranioplasty in which the defect is closed either with the original bone or a prosthetic device. Cranioplasties typically occur days to weeks after the original craniectomy. In some patients, however, cranioplasty is not immediately possible. Without the rigid structure of the skull, these patients are at risk for developing a pressure gradient across the soft tissues of the head with respect to atmospheric pressure. Consequently, the soft tissues of the brain are susceptible to movement with relation to the falx, tentorium, and foramen magnum. When the supporting hydrostatic column of cerebrospinal fluid is altered (i.e., during a LP), brain tissue can shift as atmospheric pressure exceeds intracranial pressure. This is known as paradoxical herniation. The driving force is intracranial hypotension as opposed to intracranial hypertension. It is referred to as the “syndrome of the trephinated” or “sinking skin flap syndrome.”^5^

Some cases are provoked (i.e., in the setting of a LP or shunt placement); while others are spontaneous. Paradoxical herniation is typically encountered in the weeks to months after original craniectomy^6^ making this a potential complication seen by EPs. Paradoxical herniation results in physical compression of critical brain structures leading to headache, autonomic instability (including bradycardia), altered mental status, pupillary changes, and focal neurologic deficits. Unique physical exam findings include sinking of the skin overlying the craniectomy defect; however, not all cases will have a sunken flap.^6^

As the forces generating herniation in paradoxical herniation originate from a low pressure system, management is focused on increasing intracranial pressure. This involves positioning the patient with the head of the bed down (Trendelenburg position), hydration with crystalloid, and discontinuing diuretics or hypertonic solutions.^1^ The patient should receive supportive care for nausea and pain. Intubation and advanced airway management may be necessary in severe cases. There have been reports of cases successfully treated with addition of a blood patch, a therapeutic used in our patient. In extreme situations, patients have had to return emergently to the operating room for cranioplasty.^7^

Patients with paradoxical herniation should be admitted to the ICU and neurologic status closely monitored. Highly symptomatic patients with evidence of brainstem compression should be transferred to centers with neurosurgery consultation.^3^

## CONCLUSION

Our case highlights a rare disease entity in craniectomy patients. Unlike herniation from elevated intracerebral pressures, paradoxical herniation is due to a low-pressure intracerebral state, resulting in compression of vital brain structures. It is important to inspect the contour and shape of craniectomy flaps. Management should be aimed at increasing the intracerebral pressure.
